# Customized mandibular reconstruction plates improve mechanical performance in a mandibular reconstruction model

**DOI:** 10.1080/10255842.2016.1240788

**Published:** 2016-11-25

**Authors:** Ralf Gutwald, Raimund Jaeger, Floor M. Lambers

**Affiliations:** ^a^ Department of Oral and Maxillofacial Surgery, University Hospital of Freiburg, Freiburg, Germany; ^b^ Fraunhofer Institute for Mechanics of Materials IWM, Polymer Tribology & Biomedical Materials – Group, Freiburg, Germany; ^c^ Stryker Leibinger GmbH & Co. KG, Freiburg, Germany

**Keywords:** Mandibular reconstruction, sensitivity analysis, finite element analysis, fatigue testing, implant

## Abstract

The purpose of this paper was to analyze the biomechanical performance of customized mandibular reconstruction plates with optimized strength. The best locations for increasing bar widths were determined with a sensitivity analysis. Standard and customized plates were mounted on mandible models and mechanically tested. Maximum stress in the plate could be reduced from 573 to 393 MPa (−31%) by increasing bar widths. The median fatigue limit was significantly greater (*p* < 0.001) for customized plates (650 ± 27 N) than for standard plates (475 ± 27 N). Increasing bar widths at case-specific locations was an effective strategy for increasing plate fatigue performance.

## Introduction

Tumors of the mandible often require resection of the mandible and are associated with surgical morbidity and difficulties in mastication and speaking. Mandibular reconstruction plates that bridge the resection are used to improve a patient’s quality of life by restoring the masticatory function and maintaining facial esthetics. Complications related to the removal of a mandibular reconstruction plates occur in 5–47% of all cases and are mainly due to plate exposure, infection, and tissue necrosis (Shibahara et al. 2002; Lopez et al. 2004; Militsakh et al. 2004; Ettl et al. 2010; Maurer et al. 2010; Ciocca et al. 2013).

Incidence of plate removal due to plate fracture ranges from none (Militsakh et al. 2004), 3% (Lopez et al. 2004), 4% (Maurer et al. 2010), 7% (Shibahara et al. 2002), 11% (Seol et al. 2014) to as high as 18% (Sakakibara et al. 2014). Plate fracture occurs typically in less than 6–9 months after surgery (Shibahara et al. 2002; Seol et al. 2014). Plate fractures most commonly occur on the inner curvature of a reconstruction plate where stress concentrations are located (Martola et al. 2007), and in most cases involves a resection including the mandibular angle (Shibahara et al. 2002; Sakakibara et al. 2014). Plate fracture is more frequent in patients without bone grafting (Shibahara et al. 2002; Ettl et al. 2010) and has not specifically been reported for patients with non-union of bone grafts (Rashid et al. 2012; Guerrier et al. 2015). Furthermore, plate fracture does not seem to depend on patient age or gender (Seol et al. 2014).

To avoid plate failure, plates should closely match the three-dimensional shape of the mandible. Virtual pre-operative planning of a mandibular reconstruction with stereolithographic models of the mandible and pre-operative bending of standard plates improve contouring of the mandible, and could reduce operating time and lead to a better surgery outcome (Kernan & Wimsatt 2000; Toro et al. 2007; Roser et al. 2010; Vakharia et al. 2012; Bianchi et al. 2013; Ciocca et al. 2013; Metzler et al. 2014). A further way to prevent plate fracture is to reduce stress concentrations by increasing bar widths. However, more rigid plates with wider bars are more complicated to contour. Plate failure was considered to be due to the lack of flexibility of the distal part of a plate spanning a lateral defect, complicating contouring of the mandible (Lopez et al. 2004). Thus, there is a trade-off between malleability and rigidity. To further evade plate fracture, mechanical stability should be granted through increasing bar widths at case-specific locations only.

Finite element analysis provides biomechanical information useful for implant shape optimization (Lovald et al. 2010) and comparison of different plating systems (Boyd et al. 2008). Finite element based sensitivity analyses can define the most effective locations for altering the geometry in order to reduce stress concentrations. Therefore, a sensitivity analysis provides an effective tool to determine which bar widths should be increased to most effectively strengthen a mandibular reconstruction plate.

Mechanical testing (cyclic loading or overloading) of implants, which are mounted on a synthetic or cadaveric bone, enables the biomechanical evaluation of implants in terms of yield, stiffness, and fatigue properties (Haug et al. 2001, 2002; Peterson et al. 2005; Schupp et al. 2007; Madsen et al. 2008; Gateno et al. 2013). Thus, biomechanical testing can determine the effect of a geometry change on the biomechanical performance of the plate.

The overall aim of this study was to improve the biomechanical performance of mandibular reconstruction plates by matching the implant shape to the geometry of a synthetic mandible model and strengthening plates at locations shown to be most efficient. The specific goals of this study were (1) to assess optimal locations for plate strengthening based on a sensitivity analysis which was performed for a standard mandibular reconstruction plate, (2) to determine the fatigue performance of standard plates bent by a surgeon and customized mandibular reconstruction plates by mechanical testing, and (3) to compare the location of plate failure induced by mechanical testing and the location of stress concentrations determined by finite element modeling.

## Materials and methods

### Finite element analysis

**Figure 1.**
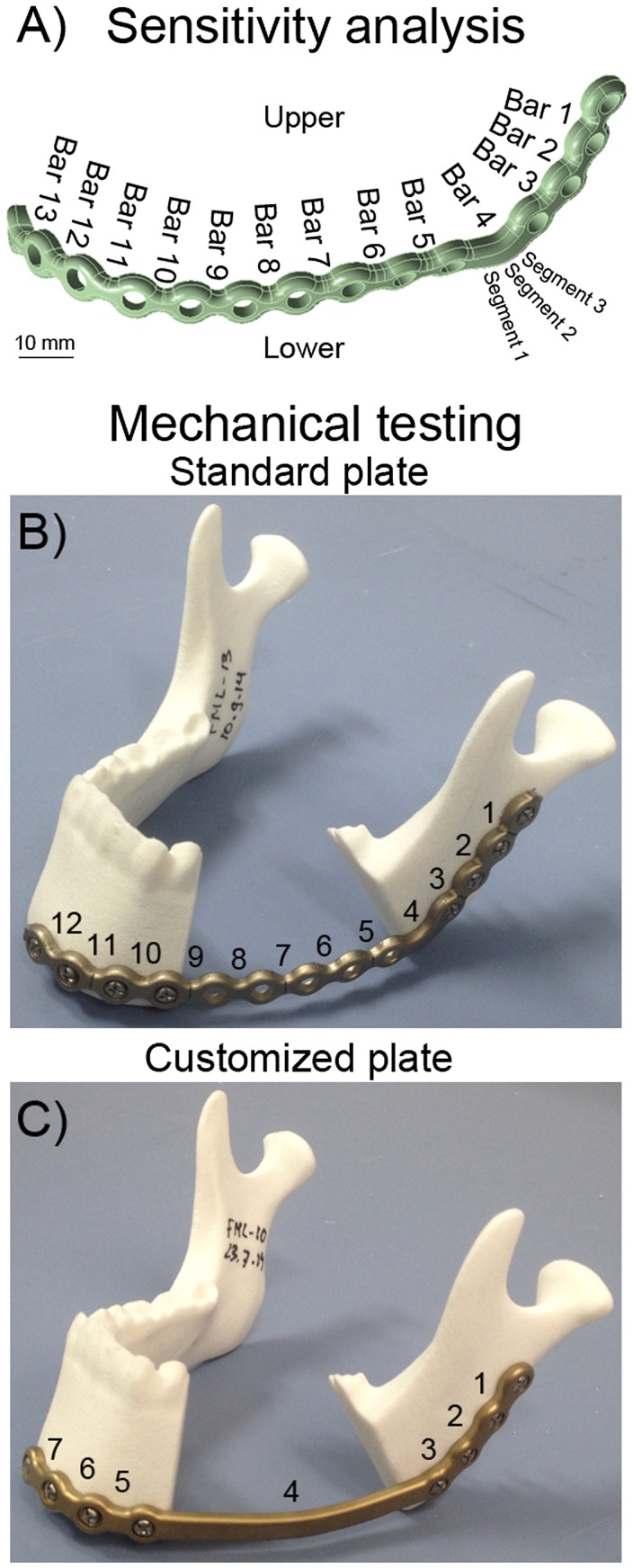
(A) The standard mandibular reconstruction plate with all bars numbered showing locations that were analyzed for the sensitivity analysis. (B) Standard mandibular reconstruction plate mounted on a synthetic mandible with a resection located between bar 4 at the angle and bar 9 at the parasymphysis. (C) Customized mandibular reconstruction plate mounted on a synthetic mandible with bar 4 spanning the resection.

**Figure 2.**
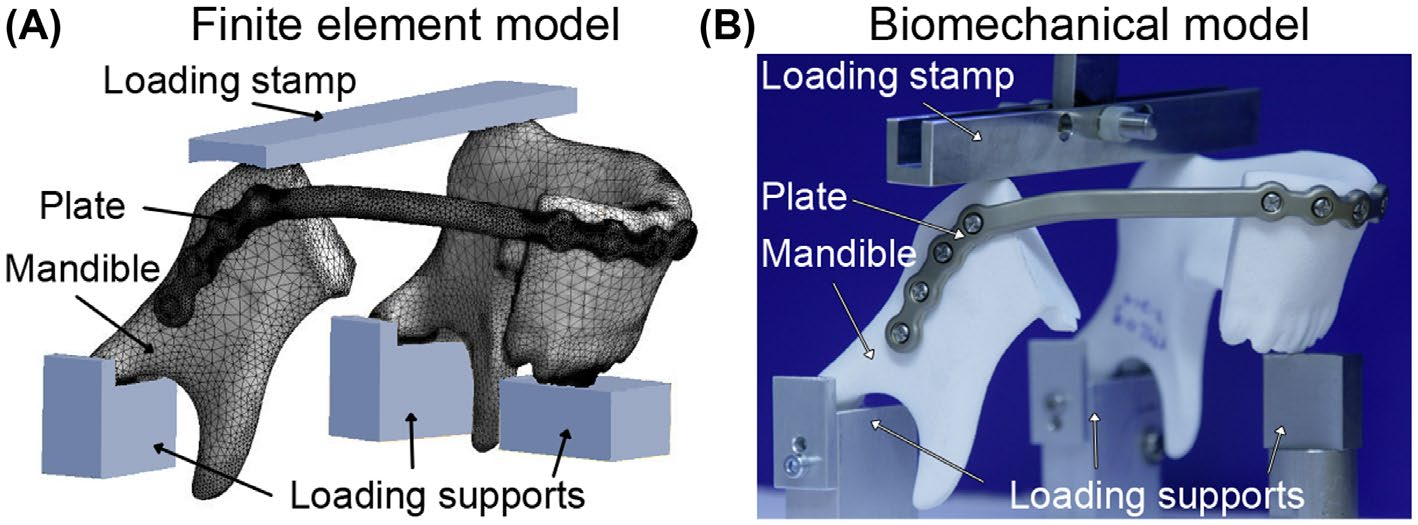
Set-up of finite element model and biomechanical loading configuration.

All finite element and sensitivity analyses were performed by CADFEM (CADFEM GmbH, Grafing near Munich, Germany) using ANSYS® Version 15.0 (ANSYS, Inc., Canonsburg, PA, USA) and OptiSLang® (Dynardo, Weimar, Germany). A three-dimensional mesh of a standard reconstruction file was constructed (PTC Creo Parametric) with all bar widths parameterized and numbered (Figure 1(A)). Further input files consisted of simplified screws (modeled as cylinders), a mandible with a resection between the last molar and the second bicuspid, and supports to closely mimic the loading configuration during biomechanical tests (Figure 2). The full model consisted of approximately 1 million elements, with a denser mesh at critical locations. Materials were assigned a Poisson’s ratio of 0.3 and a Young’s modulus of 10 Gpa for bone (which is in the range of what others have reported previously (Odin et al. 2010; Guerrier et al. 2015)), 110 Gpa for plate and screws (titanium), and 70 Gpa for the supports and loading plate (aluminum). The upper plate was displaced to transfer a load of 600 N for 1 s on the inferior side of the mandible (in *z*-direction). The displacement was constrained in *x* and *y* direction for the loading bar and in all directions for the three lower supports. No relative motion (rigid fixation) between the plate and screws (locking screws) or between bone and screws was allowed. For the interface between bone and supports a friction coefficient of 0.2 was used. The sensitivity analysis included 76 plate designs with the design variable bar width varying between 5.5 and 6.5 mm A plate configuration with bar widths of 5.5 mm and four plate designs with thicker bars at locations that contributed most to the scatter of maximum stress (i.e. have the greatest effect on reducing stress) were chosen for comparison of the maximum stresses at each inferior and superior side of each bar.

Using a similar setup, finite element analysis was performed for the plates used for mechanical testing. The analysis was performed with rigid contacts between the plate and screws and additionally with frictionless contacts between the fourth screw hole and the plate. Maximum stresses at each bar were determined. For the simulation with frictionless contacts also the reaction forces of supporting blocks and screws were assessed.

### Biomechanical testing

Fatigue performance can be expressed as the median fatigue limit (MFL). The MFL is defined as the load at which half of the samples will presumably fail and the other half of the samples will withstand the load without breaking for a given number of cycles. Mechanical tests to determine the MFL were performed on a mechanical testing machine (858 MiniBionix II, MTS, Eden Prairie, MN, USA) by the Fraunhofer Institute for Mechanics of Materials IWM (Biomedical Materials & Implants – Group, Freiburg, Germany). Standard mandibular reconstruction plates (*N* = 7, 2.8 mm profile height, bar widths of 5.5 mm at angle and 4.3 mm elsewhere, Ref. No. 55-28922, grade 2 titanium, Stryker Leibinger GmbH & Co. KG, Freiburg, Germany) were bent into shape by two experienced surgeons. Surgeons were provided with plate cutting and bending tools and a bending guideline to ensure no over-bending/damage occurred. Customized mandibular reconstruction plates (*N* = 6, 2.8 mm profile height, bar widths of 6.5 mm at angle and 5.5 mm elsewhere, Ref. No. 78-20028-1, grade 2 titanium, Stryker Leibinger GmbH & Co. KG, Freiburg, Germany) following the same contour as the standard mandibular reconstruction plate, but without screw holes bridging the resection, were planned using the proprietary BluePrint software (Stryker CMF, Kalamazoo, USA). Bar strengthening for the customized mandibular reconstruction plates was based on the sensitivity analysis described above. The mandibular reconstruction plates were fixated on synthetic mandible models (glass-sphere-filled Polyamid-12-Powder (PAGF), laser sintered, same geometry as used for finite element analysis, Speedform AG, Germany) with 4 locking screws on either mandible segment (diameter 2.7 mm, length 18 mm, Ref. No. 50-27518, grade 5 titanium, Stryker Leibinger GmbH & Co. KG, Freiburg, Germany) using drilling guides for a reproducible positioning of the plates onto the mandible models (Figure 1). During mechanical testing, the mandible models were supported below both condyles and underneath the incisors. The load was applied by a bar pressing on the angles of the mandible (Figure 2(B)) in analogy to the experimental setup described by Schupp et. al. (2007). In the aforementioned setup, the load distribution between resected and intact side of the mandible had been 30%/70%. For the current setup the load application needed to be modified to a 50%/50% distribution in order to induce plate failure. Cyclic loading was performed in load control at 3 Hz, with a 1 kN cross-head load cell (MTS). If the maximum deflection exceeded the value recorded after 1000 cycles by 1 mm or a visible crack in the plate was detected, the specimen was considered as failed. The MFL was determined according to the staircase method of Dixon and Mood (1948): if a sample failed, the maximum load was decreased by 50 N for the next sample, and if a sample survived 250,000 cycles without failing, the maximum load was increased by 50 N for the next sample.

## Results

**Figure 3.**
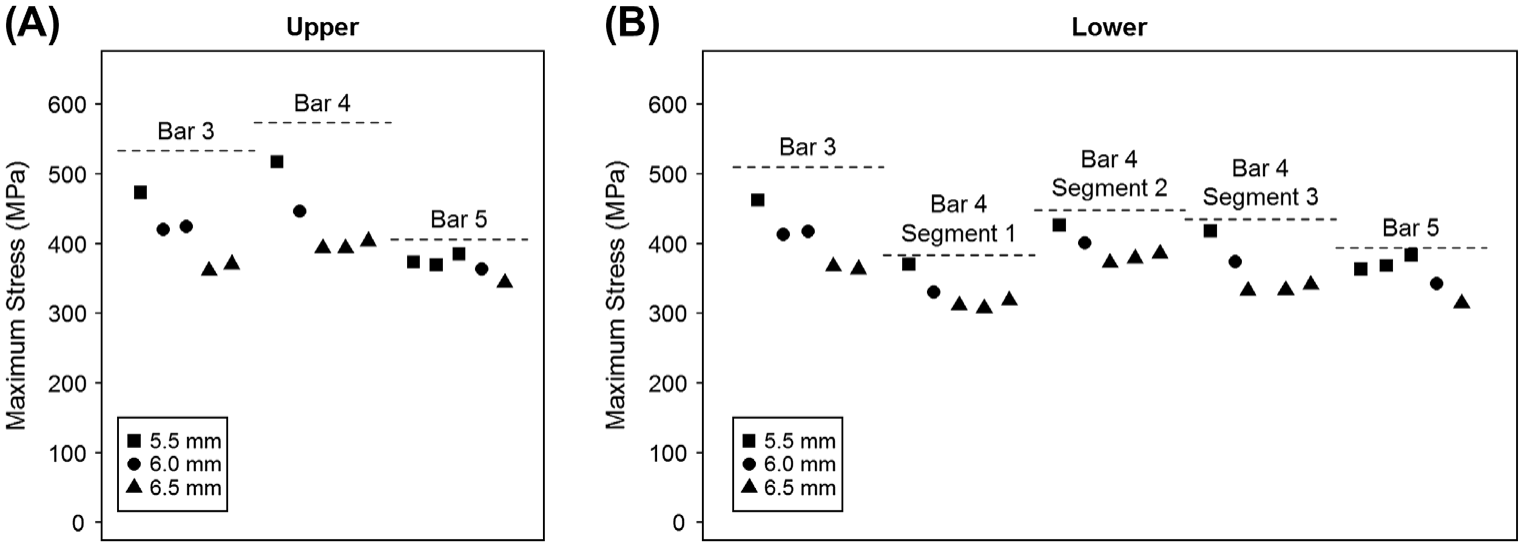
Maximum stress in bars 3, 4, and 5 at (A) the upper (superior) side and (B) the lower (inferior) side for the 5 plate configurations selected from the sensitivity analysis. The bar width is only indicated for the selected bar, because effects of plate strengthening are mostly confined to reducing stress in the bar itself and not the adjacent bars.

The results of the sensitivity analysis showed that the maximum stress was located on the superior side of bar 4 (517 MPa), and could be reduced to 393 MPa by applying plate strengthening. Stresses above 400 MPa were present in bars 3 and 4 and directly decreased if bar width was increased from 5.5 to 6.5 mm (Figure 3). Stresses above 300 MPa occurred in bars 3–5, 9, and 10, but only in bars 3–5 could the maximum stress be reduced by more than 10% and up to 32% (Table 1).

**Table 1.** Maximum stress in lower and upper bar segments and percent difference compared to the maximum stress for the design of experiments (DoE) is shown for the five designs.

		Max (DoE)	Design A		Design B		Design C		Design D		Design E	
	Bar 3		5.5 mm		6.0 mm		6.0 mm		6.5 mm		6.5 mm	
	Bar 4		5.5 mm		6.0 mm		6.5 mm		6.5 mm		6.5 mm	
	Bar 5		5.5 mm		5.5 mm		5.5 mm		6.0 mm		6.5 mm	
	Bars 1, 2, 6–13		5.5 mm		5.5 mm		5.5 mm		5.5 mm		5.5 mm	
		(MPa)	(MPa)	(%)	(MPa)	(%)	(MPa)	(%)	(MPa)	(%)	(MPa)	(%)
Bar 1	Upper	102	71	−31	75	−26	79	−22	82	−20	83	−18
	Lower	128	100	−22	105	−18	105	−18	109	−15	113	−12
Bar 2	Upper	308	258	−16	270	−12	279	−9	286	−7	293	−5
	Lower	264	225	−15	239	−9	245	−7	243	−8	258	−2
Bar 3	Upper	533	473	−11	420	−21	424	−20	361	−32	370	−31
	Lower	510	462	−9	413	−19	417	−18	367	−28	363	−29
Bar 4	Upper	573	517	−10	446	−22	393	−31	393	−31	403	−30
	Lower segment 1	383	370	−3	330	−14	311	−19	307	−20	318	−17
	Lower segment 2	448	426	−5	401	−10	372	−17	378	−16	385	−14
	Lower segment 3	435	418	−4	374	−14	332	−24	333	−23	341	−22
Bar 5	Upper	406	373	−8	369	−9	385	−5	363	−11	343	−16
	Lower	394	363	−8	368	−7	383	−3	342	−13	314	−20
Bar 6	Upper	200	179	−10	170	−15	184	−8	190	−5	196	−2
	Lower	227	193	−15	202	−11	207	−9	212	−7	217	−4
Bar 7	Upper	100	77	−23	83	−17	89	−11	93	−7	99	−1
	Lower	145	102	−29	118	−19	125	−14	128	−11	135	−7
Bar 8	Upper	205	194	−6	202	−2	193	−6	186	−9	189	−8
	Lower	182	176	−3	179	−2	171	−6	165	−10	167	−8
Bar 9	Upper	408	387	−5	399	−2	386	−5	381	−7	386	−5
	Lower	358	336	−6	355	−1	343	−4	334	−7	338	−5
Bar 10	Upper	324	307	−6	320	−1	315	−3	304	−6	310	−5
	Lower	317	315	−1	323	2	321	1	315	−1	319	1
Bar 11	Upper	67	63	−6	63	−6	64	−4	64	−5	64	−4
	Lower	64	56	−13	49	−24	53	−18	54	−16	55	−14
Bar 12	Upper	43	40	−7	42	−2	39	−10	37	−13	37	−13
	Lower	58	52	−9	56	−4	53	−9	51	−12	51	−11
Bar 13	Upper	26	26	0	27	1	25	−4	24	−8	25	−7
	Lower	33	31	−4	33	2	32	−3	31	−6	31	−5

Note:

Bars with a stress above 300 MPa and a reduction in maximum stress greater than 10% when compared to DoE are highlighted in red.

**Figure 4.**
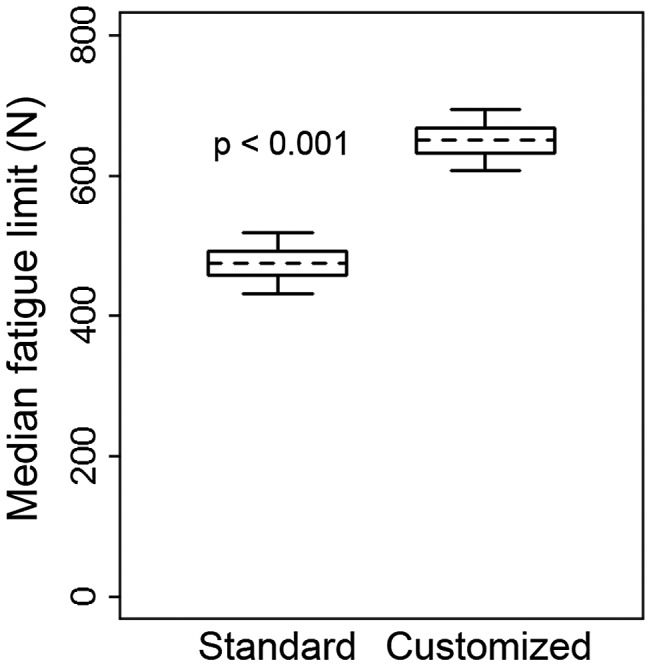
The median fatigue limit for standard (left) and customized (right) mandibular reconstruction plate. Box plot shows a box between the 25th and 75th percentile, a dotted line at the mean, and whiskers extending to the 5th and 95th percentile based on a normal (Gaussian) distribution.

**Figure 5.**
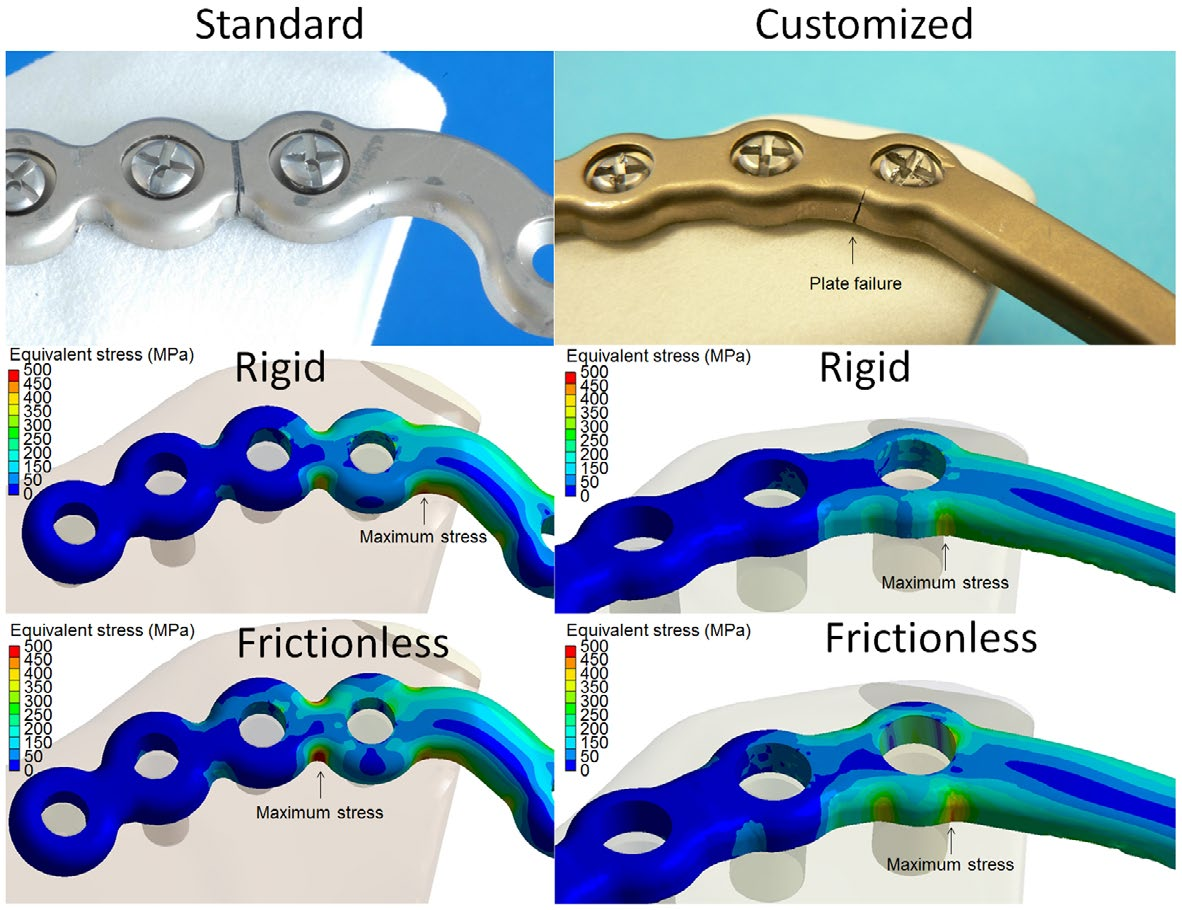
Location of plate failure (upper) and maximum stress (lower) in standard (left) and customized (right) mandibular reconstruction plates.

The fatigue performance determined by mechanical testing was significantly greater (*p* < 0.001, unpaired *t*-test) for customized mandibular reconstruction plates (MFL = 650 N ± 27 N) than for standard reconstruction plates (MFL = 475 N ± 27 N, Figure 4). For standard mandibular reconstruction plates failure occurred at 550 N, run-outs (surviving 250,000 cycles) at 400 N, and both failure and run-outs at 450 and 500 N (Table 2). For customized mandibular reconstruction plates, failure occurred at 700 N, run-outs at 600 N, and both failure and run-outs at 650 N (Table 3). Plate fracture occurred at the superior side of the plate just posterior to the resection between the third and fourth screw hole for both standard and customized plate (Figure 5).

**Table 2.** The number of cycles to failure is indicated for the standard mandibular reconstruction plates. A run-out (r.o.) indicates that the displacement limit was not reached within 250,000 cycles.

		Sample						
*F*_max_ (N)	*F*_min_ (N)	1	2	3	4	5	6	7
700	70							
650	65							
600	60							
550	55					22,309		
500	50				r.o.		90,458	
450	45	66,710		r.o.				r.o.
400	40		r.o.					

**Table 3.** The number of cycles to failure is indicated for the customized patient-specific mandibular reconstruction plates. A run-out (r.o.) indicates the displacement limit was not reached within 250,000 cycles.

		Sample					
*F*_max_ (N)	*F*_min_ (N)	1	2	3	4	5	6
700	70					55,000	
650	65		115,724		r.o.		r.o.
600	60	r.o.		r.o.			
550	55						
500	50						
450	45						
400	40						

The location of plate failure was in agreement with the location of greatest stress for the standard plate if frictionless contact was assumed between the fourth screw hole and the plate (Figure 5). The location of plate failure in the customized plate did not match the location of maximum stress regardless of the modelling of the contacts, but did occur at the second greatest stress concentration (Figure 5). The maximum stress was 37% greater for the standard plate (509 MPa) than for the customized plate (372 MPa) if a rigid contact was simulated and 22% greater if a frictionless contact was simulated (Table 4).

**Table 4.** Maximum stresses in standard and customized patient-specific mandibular reconstruction plates in the bars at resections.

		Standard				Patient-specific			
		Width (mm)	Rigid stress (MPa)	Frictionless stress (MPa)	Frictionless/rigid (%)	Width (mm)	Rigid stress (MPa)	Frictionless stress (MPa)	Frictionless/rigid (%)
Bar 1	Upper	4.3	<100	<100	n/a	5.5	<100	<100	n/a
	Lower		<100	<100	n/a		<100	<100	n/a
Bar 2	Upper	4.3	<100	146	180	6.5	<100	<100	n/a
	Lower		120	169	141		<100	<100	n/a
Bar 3	Upper	4.3	432	577	134	6.5	205	389	190
	Lower		452	614	136		213	399	187
Bar 4	Upper	5.5	509	470	92	6.5	372	502	135
	Lower		387	391	101		357	407	114
Bar 5	Upper	4.3	389	369	95	5.5	132	135	102
	Lower		398	380	95		116	112	97
Bar 6	Upper	4.3	170	164	96	5.5	<100	<100	n/a
	Lower		191	180	94		<100	<100	n/a
Bar 7	Upper	4.3	<100	<100	n/a	5.5	<100	<100	n/a
	Lower		<100	<100	n/a		<100	<100	n/a
Bar 8	Upper	4.3	167	171	102				
	Lower		160	165	103				
Bar 9	Upper	4.3	n/a	n/a	n/a				
	Lower		349	353	101				
Bar 10	Upper	4.3	232	235	101				
	Lower		220	222	100				
Bar 11	Upper	4.3	<100	<100	n/a				
	Lower		<100	<100	n/a				
Bar 12	Upper	4.3	<100	<100	n/a				
	Lower		<100	<100	n/a				
Bar 13	Upper	4.3	<100	<100	n/a				
	Lower		<100	<100	n/a				

Note: Stresses above 300 MPa are shown in red.

## Discussion

Complications after mandibular reconstructive surgery can be debilitating for a patient and need to be minimized. One option for reducing complications is avoiding plate failure though improving biomechanical properties of mandibular reconstruction plates. The current study shows that the fatigue performance of a customized mandibular reconstruction plate which was 3D shaped in a dedicated pre-operative manufacturing process is significantly greater than the fatigue performance of a standard intra-operatively manually-bent mandibular reconstruction plate. Our results indicate that individual virtual planning and optimization of the plate design provide effective strategies for improving the biomechanical performance of mandibular reconstruction plates.

There are a number of strengths to the current study. First, customized plates were planned with novel software, allowing three-dimensional plate planning, taking mandible anatomy (e.g. nerves, roots) into account, and enabling strengthening bars at individual locations. Second, sensitivity analyses determined the most optimal locations for plate strengthening, thereby taking all possible arrangements of bar widths into account. Third, our analyses of biomechanical competence include empirical (mechanical) testing and computational (finite element) analyses, confirming the benefit of plate strengthening in two separate ways.

There are some limitations that must be considered when interpreting our results. First, just one type of resection was taken into account in this paper. The resection was chosen at the angle due to its frequent prevalence (Shibahara et al. 2002; Coletti et al. 2009; Ettl et al. 2010) and because a plate fracture was most frequently observed with such lateral defects (Katakura et al. 2004). It is possible that in other arrangements plate strengthening is more beneficial at other locations, although further analyses (unpublished data) suggest that, in general, plate strengthening is most effective at posterior (close to the angle) rather than the anterior (close to the symphysis) resection site. In contrast, a previous study reported that the stress was the greatest in a screw located closest to the resection at the side of load application at the parasymphysis, indicating that stresses in the plate were greatest in the frontal area (Bujtar et al. 2014). This discrepancy is likely due to the simulation of a bilateral loading in the current study vs. unilateral bite forces in the previous study. Second, the loading configuration for biomechanical testing and finite element analysis was a simplification of *in vivo* muscle and biting forces. In agreement with a previous study that assessed mechanical performance of mandibular reconstruction systems (Schupp et al. 2007), the condyles and incisors were supported and a load was applied at the angles. To ensure plate failure in the current study, the mechanical testing setup had to be modified. While in the previous setup the load distribution between the resected and intact side of the mandible was 30 vs. 70%, the load distribution was transmitted equally between sides in the current study to increase the load supported by the plate. Furthermore, while in the previous study a maximum load of 300 N was applied and caused plate failure, a load of at least 450 N was required for failure of the standard reconstruction plate tested in this study. This inconsistency could be due to differences in plate strength, mandible model, or resection (although it was attempted to match the resection as close as possible). Similar boundary conditions as used in the current finite element analysis have been used by other research groups. Several plating systems have been assessed using a finite element model where both condyles are constrained, a molar is constrained unilaterally, and muscle forces are simulated as vectors attaching to multiple points at the angle and ramus (Lovald & Khraishi 2007; Boyd et al. 2008; Lovald et al. 2010; Bujtar et al. 2014). Furthermore, comparable mechanical testing setups have been reported with modifications such as supporting the dental arch instead of the incisors (Gateno et al. 2013) or tensile loading at the incisal edge and fixing the ramus region with a steel rod (Haug et al. 2001; Peterson et al. 2005; Madsen et al. 2008). Mimicking the complex three-dimensional loading configuration of the mandible is challenging and a simplification facilitates reproducible testing and a comparison of specific design variables with consistent outcomes. Therefore, in line with previous studies, the current test setup allows meaningful comparisons between mandibular reconstruction plates.

The mechanical performance was compared between a standard reconstruction plate with screw holes spanning the defect and a customized plate with a bar spanning the defect. The dissimilarity in number of screw holes complicates distinguishing the effect of bar width vs. general plate design on the mechanical performance. Since stress concentrations are often located at screw holes, it is intuitive that a plate design with a bar rather than screw holes spanning a defect should result in a lower maximum stress. The sensitivity and finite element analysis showed that maximum stress could be reduced by 31% by changing just bar width, while the maximum stress in the standard plate was 37% greater than for the customized plate (using the same boundary conditions). It follows that if no bone grafts are used during reconstruction, just having a bar seems more stable, and an advantageous option in customized plate planning.

While the current study described stresses in a mandibular reconstruction plate without the use of bone grafts (secondary reconstruction), the results can be translated to reconstruction cases that include bone grafts (primary reconstruction). Bone grafts and the mandible grow together (heal) in about 2–3 months after surgery (Marx 2007), but until then, forces are transmitted through the plate. Therefore, forces transmitted through the plate in a primary reconstruction initially equal those of a secondary reconstruction. Reasons for performing an analysis without bone grafts are that the specification of boundary conditions between mandible and graft in finite element analysis is difficult and the preparation of models for mechanical testing is more complicated. Furthermore, grafts vary considerably in size and shape, especially because autologous bone grafts may be harvested from the fibula, iliac crest, radial forearm, or scapula (Goh et al. 2008).

A comparison between stresses in the plate based on a linear finite element analysis and the yield strength (permanent deformation) or ultimate strength (failure) of the material should be made with caution. A linear finite element analysis assumes constant stiffness even when deformation occurs (which is a simplification of reality). Furthermore, load redistribution and relaxation/stress hardening effects that occur when a material is dynamically loaded are not considered in a finite element analysis that simulates a single load. Thus, discrepancies may exist between absolute stress values/strength estimation from linear finite element analysis and empirically derived strength. Non-linear finite element analysis provides a better agreement between simulated and actual material stresses, but is computationally expensive and more complex. In the current finite element analysis a load of 600 N was simulated and lead to multiple elements experiencing a stress above the yield strength (275 MPa for titanium grade 2) and even above the ultimate strength (345 MPa for titanium grade 2), indicating plate failure. Nevertheless, the load at which only half of the customized plates failed (MFL) was 650 N as assessed with mechanical testing. While the comparison of absolute stress values between linear finite element analysis and the material properties may not be suitable, the locations of maximum stresses can be compared. The incidence of plate failure between two screw holes at the posterior side of the resection is consistent with a previous study where plate fracture occurred between two screw holes in a plate bridging a lateral defect (Lopez et al. 2004) and in analogy to the observation that the stress in a reconstruction plate was the greatest at the osteotomy site (Boyd et al. 2008). Nevertheless, plate failure in similar defects has also been observed between screw holes spanning the resection and between screw holes at the transition between the resected area and the distal bone segment (Martola et al. 2007; Schupp et al. 2007). Likely, the location of plate failure depends on the loading configuration and plate design, where some designs are more effective at deflecting stress concentrations than others.

Although the current study did not examine bite forces, it is useful to consider how the plate strength compares to the forces it may be subjected to. The MFL of customized mandibular reconstruction plates (650 N) is above the range of mean physiological bite forces reported in literature (130–626 N), although the maximum bite force of a healthy person depends on sex, age, and method of measurement (Tate et al. 1994; Wedel et al. 1994; Ellis et al. 1996; Harada et al. 2000; Iwase et al. 2006). Bite forces in patients undergoing mandibular surgery are about half of the bite forces of controls pre-operatively, return to the preoperative level 6 months after surgery, and return to the level of healthy subjects about 2–3 years after surgery (Ellis et al. 1996; Harada et al. 2000). Therefore, the MFL of the standard reconstruction plate (475 N) is in the higher range of physiological bite forces in patients after mandibular surgery.

The current study only assessed the stresses in the plate and not in the screws or the mandible, because the elements of the mandible and screws were not fine enough. Detailed simulation is required in order to provide reliable information on stresses in screws; peak stresses were about 3-fold in fully threaded screw compared to a simple cylinder in a finite element model (Bujtar et al. 2014). Furthermore, boundary conditions affect the location and magnitude of stresses. The observation that the location of the maximum stress in the finite element analysis and plate failure during mechanical testing coincided for the standard plate only if frictionless contact was assumed, indicated that loosening of the screw might have occurred during mechanical testing. During mandibular reconstruction, stresses are carried by the plate over the resection and are transferred back to the bone with the screws. Therefore, if greater loads are taken by the plate, also the screws are subjected to greater loads. In analogy, increasing screw diameter is effective at reducing stresses (Chaudhary et al. 2008). The location of plate failure in the customized plate did not match the location of highest stress during finite element analysis regardless of how the contact between the fourth screw and plate was modeled. Possibly a slight alteration in the loading mode caused the difference. In the finite element analysis, the load transferred through the intact side was 231 vs. 191 MPa on the resection side (55%/45%), while during mechanical testing the load distribution had been approximately 50%/50%. Other reasons for the inconsistency in failure location include the analysis of a single load in the finite element analysis vs. cyclic mechanical loading, material property assumptions, and the simplified representation of screws. It should be noted, however, that failure occurred at the second greatest stress location assessed by the finite element analysis.

Several methods are available for assessing implant shapes with the most optimal mechanical performance. Mechanical testing enables comparison of different plating or locking systems (Peterson et al. 2005; Schupp et al. 2007; Madsen et al. 2008) and multiple finite element analyses in which specific parameters (e.g. bar width) or implant shapes are varied enable defining optimal designs (Fernandez et al. 2003; Knoll et al. 2006; Nagasao et al. 2010; Qin et al. 2015). For example, stresses could significantly be reduced by increasing the radius of the fillet from 0 to 1 mm, but further increasing the radius had no additional benefit (Qin et al. 2015). A similar but more thorough and automated version of multiple finite element analyses with variation in specific parameters was presented in the current study. Although results of this study are intuitive it is novel in its use of a sensitivity analysis that enables deriving information equivalent to over 100 FE simulations. A sensitivity analysis can determine which design parameters have the greatest benefit on the complete structure, and the design parameters can be set such that manufacturing processes can be maintained. If there are no constraints on plate shape, such as with additive manufacturing technologies, topological optimization represents a refined method. In topological optimization, parts of a volume that do not contribute to overall stiffness are removed until only the most essential parts that maximize the plates resistance remain (down to a predetermined volume). The maximum stress in a bone plate for fracture fixation of the mandible was about one third lower when the implant shape was based on topological optimization than when fixated with two miniplates with similar volume (Lovald et al. 2010). In future, combined topological optimization and sensitivity analysis may be performed to create novel implant shapes with optimal screw hole positions. Of note, finite element analyses contribute to assessing the mechanical performance of implants, but shape limitations due to anatomy, handling or approachability are not considered.

The plate fatigue performance could further be enhanced by increasing the bar width and plate profile height, but the benefit of an overdesigned plate (stronger than the forces it is subjected to) is questionable for several reasons. First, a plate with even wider bars and/or higher profile will be less malleable, thus complicating close contouring if intra-operative modifications are necessary. Second, if all stresses are carried by a plate and consequently little forces are transmitted through the bone, bone resorption could occur (a phenomenon called stress shielding) within 10 weeks of implant fixation (Perren 1979; Uhthoff et al. 2006). Third, if greater stresses are transmitted through plates, greater stresses will be transferred through screws; therefore, introducing the danger of screw breakage if screw strength is not simultaneously improved. In effect, screw breakage rather than plate failure, has been observed in the clinic (Siegmund et al. 2000). In the current analysis the reaction stresses at the screws were already up to 400 MPa.

Although the costs of customized implants exceed the costs of standard plates, the use of customized implants is financially viable. The use of preoperative planning can considerably reduce operating room time. Since 30–40% of hospital expenses are accountable to operating room costs, the price of implants is easily offset by time savings (Kernan & Wimsatt 2000). Depending on the skill and routine of the surgeon, preoperative planning and the use of stereolithographic models can reduce operation by about 1–1.5 h (Toro et al. 2007; Zweifel et al. 2015). One minute in the operation room during a mandibular reconstruction costs between 48 and 103 dollars, depending on the hospital (Haddock et al. 2012; Rustemeyer et al. 2014). Furthermore, in several countries reimbursement fees provided by statutory health insurance covered 172% of the costs (Rustemeyer et al. 2014). Thus, although equipment for virtual planning is more expensive, costs can be regained through saving surgery time or from reimbursement. Furthermore, patient outcome might be better due to the shorter operating time, better bony contact between grafts (more precise controlling) and (probably) lower chance for recurrence.

In conclusion, we have demonstrated that customized mandibular reconstruction plates have a better biomechanical performance than manually bent stock reconstruction plates. Adapting the implant shape to a patient’s anatomy by optimal contouring, along with the ability to strengthen the plate at locations determined by the surgeon, may avoid hardware-related complications in mandibular reconstructions.

## Disclosure statement

RG does consultancy for Stryker, RJ received a research grant from Stryker, and FML has commercial interest.

## Funding

This work was sponsored by the Stryker Leibinger GmbH & Co. KG.
